# Missense mutation of angiotensin converting enzyme gene in an Alzheimer’s disease patient: a case report

**DOI:** 10.3389/fnins.2024.1343279

**Published:** 2024-03-07

**Authors:** Mingyue He, Fan Zhang, Jing Qi, Wei Zhang

**Affiliations:** ^1^Department of Neurology, Beijing Tiantan Hospital, Capital Medical University, Beijing, China; ^2^Center for Cognitive Neurology, Department of Neurology, Beijing Tiantan Hospital, Capital Medical University, Beijing, China; ^3^China National Clinical Research Center for Neurological Diseases, Beijing Tiantan Hospital, Capital Medical University, Beijing, China; ^4^Center of Parkinson's Disease, Beijing Institute for Brain Disorders, Beijing, China; ^5^Beijing Key Laboratory on Parkinson Disease, Beijing, China

**Keywords:** Alzheimer’s disease, angiotensin converting enzyme, missense mutation, early-onset, neuropsychiatric symptoms, rapid progression

## Abstract

Alzheimer’s disease (AD) is the most common type of cognitive impairment in the elderly. In this report, we presented a case of a 52-year-old woman with rapid disease progression within 6 months. She was diagnosed with mild dementia according to the clinical symptoms and neuropsychological assessment results. Based on the results of neuropathological proteins in cerebrospinal fluid, cranial magnetic resonance imaging, and positron emission tomography/computed tomography, the patient showed the presence of β amyloid deposition, pathologic tau along with neurodegeneration [A+T+(N+)], indicative of AD. Whole exome sequencing revealed a heterozygous C-to-T missense mutation of nucleotide 3,755 (c.3755C > T) in exon 25 of the angiotensin converting enzyme (ACE) gene on chromosome 17q23 (rs762056936).

## 1 Introduction

Alzheimer’s disease (AD) is the most common type of cognitive impairment in the elderly, characterized by the decline of multiple cognitive domains, varying degrees of neuropsychiatric symptoms and compromised activities of daily living (ADL) (Scheltens et al., 2021). Depending on the age of disease onset, AD is generally divided into early-onset AD (EOAD) and late-onset AD, in which EOAD usually occurs before the age of 65 and accounts for 5–10% of all AD cases (Zhu et al., 2015). Gene mutations are more common in EOAD than in late-onset AD, and the most frequent culprit genes include amyloid precursor protein (APP), presenilin 1 (PSEN1) and presenilin 2 (PSEN2)(Dai et al., 2018).

In recent years, it has been reported that angiotensin converting enzyme (ACE) gene mutations, such as insertion/deletion (I/D) polymorphisms, are associated with increased susceptibility to AD (Yuan et al., 2015; Xin et al., 2021). In this paper, we report for the first time a new type of missense mutation of the ACE gene in an EOAD patient with rapid disease progression and review the research progress of the ACE gene and AD.

## 2 Case presentation

A 52-year-old woman was admitted to the Center for Cognitive Neurology, Department of Neurology, Beijing Tiantan Hospital, Capital Medical University on April 27, 2022. Six months ago, the patient developed hallucination and delusion without obvious causes. She suspected that people on TV were spying on her family members and that her pension had been stolen by strangers. Additionally, she had visual hallucination of seeing non-existent persons who taught her how to cook. Two months ago, her hallucination and delusion alleviated spontaneously, but she began exhibiting recent memory decline indicated by forgetting what had just happened and forgetting to cook. Furthermore, her abilities to calculate, organize and complete multitasking, and recognize family members were compromised at times. Her capacity to take care of herself in daily life was mildly compromised. She had no other neuropsychiatric symptoms, including anxiety, depression and apathy, and had no significant change in her weight, appetite, sleep, bowel movement, and urinary function. She also had no other coexisting disorders and no family history of dementia or psychiatric disorders.

### 2.1 Neurological system examination

The neurological system examination revealed that the patient had declined episodic memory, calculation, attention, executive function, and visuospatial ability. Consciousness, language, cranial nerves, muscle strength and tone, posture and gait, ataxia, sensory and tendon reflexes were all normal, and various pathological reflexes were negative.

### 2.2 Assessment scales of clinical symptoms

The patient’s global cognitive function was assessed by the Mini-Mental State Examination (MMSE) (Folstein et al., 1975) and the Montreal Cognitive Assessment (MoCA) (Nasreddine et al., 2005) scales, on which she scored 17 points and 12 points, respectively (Table 1).

**Table 1:** The scores of rating scales for the clinical symptoms of the patient.

Clinical symptom	Patient’s scores	Full scores	Sub-item
Cognitive function			
Global cognitive function			
MMSE (points)	17	30	Orientation-4, Attention and calculation-5, Recall-1, Repetition-1, Instruction-1, Drawing-1
MoCA (points)	12	30	Visuospatial and executive functions-4, Naming-1, Attention-4, Language-3, Abstraction-2, Delayed recall-3, Orientation-1
CDR (points)	1	3	/
Individual cognitive domain function			
*Memory*			
AVLT N1-3 (points)	6	36	/
AVLT N4 (points)	0	12	/
AVLT N5 (points)	0	12	/
AVLT N1-5 (points)	6	12	/
AVLT N6 (points)	0	12	/
AVLT N7 (points)	11	24	/
RCFT-delayed recall (points)	4.5	36	/
*Language*			
AFT (points)	5	/	/
VFT-H (points)	6	/	/
VFT- alternating fluency (points)	2	/	/
BNT (points)	28	30	/
*Visuospatial ability*			
RCFT-imitation (points)	22.5	36	/
*Attention / Executive function*			
TMT-A (points, seconds)	25, 92	25, /	/
TMT-B (points, seconds)	13, 240	25, /	/
SCWT-A (points, seconds)	50, 30	50, /	/
SCWT-B (points, seconds)	50, 78	50, /	/
SCWT-C (points, seconds)	15, 168	50, /	/
SDMT (points)	7	/	/
Neuropsychiatric symptom			
NPI (points)	2	144	Delusion +2
MAES (points)	5	42	/
CMAI (points)	29	203	/
HAMA (points)	4	56	/
HAMD (points)	6	78	/
PSQI (points)	3	21	/
ESS (points)	1	24	/
Activities of daily living			
ADL (points)	25	80	/

MMSE, Mini-Mental State Examination; MoCA, Montreal Cognitive Assessment; CDR, Clinical Dementia Rating; AVLT, Auditory Verbal Learning Test; RCFT, Rey-Osterrieth Complex Figure Test; AFT, Animal Fluency Test; VFT, Verbal Fluency Test; BNT, Boston Naming Test; TMT, Trail Making Test; SCWT, Stroop Color and Word Test; SDMT, Symbol Digit Modalities Test; NPI, Neuropsychiatric Inventory; MAES, Modified Apathy Evaluation Scale; CMAI, Cohen-Mansfield Agitation Inventory; HAMA, Hamilton Anxiety Scale; HAMD, Hamilton Depression Scale; PSQI, Pittsburgh Sleep Quality Index; ESS, Epworth Sleepiness Scale; ADL, Activities of Daily Living.

In terms of the functions of multiple cognitive domains, the Auditory Verbal Learning Test (AVLT) (Guo et al., 2009), and Rey-Osterrieth Complex Figure Test (RCFT)-delayed recall (Shin et al., 2006) were used to assess her memory function. The Verbal Fluency Test (VFT) (Mok et al., 2004) and Boston Naming Test (BNT) (Katsumata et al., 2015) were used to assess her language ability. The RCFT-imitation was used to assess her visuospatial ability. The Trail Making Test (TMT) (Wei et al., 2018), the Stroop Color and Word Test (SCWT) (Bondi et al., 2002), and the Symbol Digit Modalities Test (SDMT) (Fellows and Schmitter-Edgecombe, 2019) were used to assess her attention and executive function. These data indicated impairments in her immediate recall, delayed recall, cue recall, language, visuospatial ability, attention, and executive function (Table 1).

The Neuropsychiatric Inventory (NPI) scale was used to evaluate her total neuropsychiatric symptoms (Cummings et al., 1994). The individual neuropsychiatric symptoms, including apathy, agitation, depression, anxiety, sleep condition, and excessive daytime sleepiness were assessed by the Modified Apathy Estimate Scale (Starkstein et al., 1992), the Cohen-Mansfield Agitation Inventory scale (Koss et al., 1997), the Hamilton Depression Scale (Hamilton, 1960), the Hamilton Anxiety Scale (Hamilton, 1959), the Pittsburgh Sleep Quality Index (Buysse et al., 1989) and the Epworth Sleepiness (Johns, 1991), respectively. The results of these scales were shown in Table 1, which indicated that the patient had no obvious neuropsychiatric symptoms.

Furthermore, the ADL was assessed using the ADL scale (Katz et al., 1963), and disease severity was assessed using the Clinical Dementia Rating (CDR) scale (Hughes et al., 1982) (Table 1).

### 2.3 Cerebrospinal fluid (CSF)

Lumbar puncture was performed and CSF was collected to measure the potentially pathogenic variables of the patient. The results revealed that AD biomarkers, including β amyloid protein (Aβ)_1-42_ and Aβ_1-42_/Aβ_1-40_, were decreased, and phosphorylated tau (P-tau) and total tau (T-tau) were elevated. Other variables, including the oligoclonal zone, inflammatory factors, autoimmune encephalitis (AE) antibody profile, and 14–3-3 protein, showed no abnormalities (Table 2).

**Table 2:** The levels of potentially pathogenetic variables in CSF from the patient.

Items	Sample and methods	Results	Reference range (age-matched)
Initial pressure	CSF	100 mmH_2_O	80–180 mmH_2_O
Routine	CSF	Colorless, clear, WBC 1/uL	—
Biochemistry	CSF, Colorimetry	GLU 4.41 mmol/L Pro 27.86 mg/dL Cl 127 mmol/L LAC 1.60 mmol/L	2.50–4.50 mmol/L 15.00–45.00 mg/dL 118–132 mmol/L 1.10–2.40 mmol/L
AD biomarkers	CSF, ELISA	Aβ_1-42_ 151.36 pg/mL Aβ_1-40_ 7470.46 pg/mL Aβ_1-42_/Aβ_1-40_ 0.02 pg/mL P-tau 56.16 pg/mL T-tau 450.78 pg/mL	≥ 651.00 pg/mL ≥ 7000.00 pg/mL > 0.05 pg/mL ≤ 50.00 pg/mL ≤ 399.00 pg/mL
24-h intrathecal IgG synthesis rate	CSF, Immunoturbidimetric assay	Normal	—
IgG oligoclonal bands	CSF, IEF	Negative	Negative
Inflammatory factors TNF-α, IL-1β, IL-6, IL-8, IL-10, IL-2R	Blood and CSF, CLIA	Normal	—
Infection virus anti-IgM profile RUB, CMV, HSV1, HSV2, EBV, COXV, TOX	Blood and CSF, ELISA	Negative	Negative
AE antibody profile: PNMA2, Ri, Amphiphysin, Hu, Yo, CV2, NMDAR, AMPAR1, AMPAR2, CASPR2, GABABR1, GABABR2, GABAARα1, GABAARα3, GABAARβ3, GABAARγ2, LGI1, IgLON5, D2R, DPPX, GlyR, mGluR5, Neurexin3, GAD65, α3AchR	Blood and CSF, WB and CBA	Negative	Negative
14–3-3 protein	CSF, WB	Negative	Negative
Prion	CSF, RT-QuIC	Negative	Negative
Cytology	CSF	Normal	—

CSF, cerebrospinal fluid; WBC, white blood cell; GLU, glucose; Pro, protein; Cl, chloride ion; LAC, lactic acid; AD, Alzheimer’s disease; ELISA, enzyme linked immunosorbent assay; Aβ, β amyloid; P-tau, phosphorylated tau; T-tau, total tau; IEF, isoelectric focusing; TNF, tumor necrosis factor; IL, interleukin; CLIA, chemiluminescence immunoassay; RUB, rubella virus; CMV, cytomegalo virus; HSV, herpes simplex virus; EBV, Epstein–Barr Virus; COXV, coxsackie virus; TOX, toxoplasma gondii; AE, autoimmune encephalitis; PNMA2, paraneoplastic antigen Ma2; Ri (ANNA-2), anti-neuronal nuclear antibody-2; Hu (ANNA-1), anti-neuronal nuclear antibody-1; Yo (PCA-1), purkinje cell cytoplasmic antibody type1; CV2 (CRMP5), collapsin response mediator protein 5; NMDAR, N-methyl-D-aspartate receptor; AMPAR, alpha-amino-3-hydroxy-5-methyl-4-isoxazole-propionic acid receptor; CASPR2, contactin-associated protein-2; GABABR, gamma-aminobutyric acid type B receptor; GABAAR, gamma-aminobutyric acid type A receptor; LGI1, leucine rich glioma inactivated 1; IgLON5, immunoglobulin-like cell adhesion molecule 5; D2R, dopamine-2 receptor; DPPX, dipeptidyl peptidase-like protein; GlyR, glycine receptor; mGluR5, metabotropic glutamate receptor 5; GAD65, glutamic acid decarboxylase-65; α3AchR, alpha3 subunit of the nicotinic acetylcholine receptor; WB, Western Blot; CBA, cell based assay; RT-QuIC, real-time quaking-induced conversion.

### 2.4 Cranial magnetic resonance imaging (MRI)

**Figure 1:**
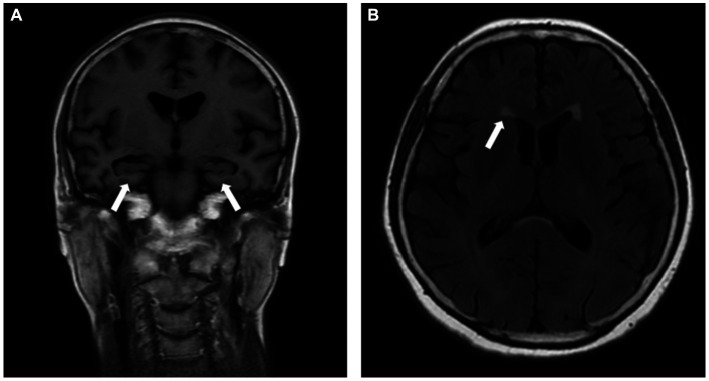
Cranial MRI showed bilateral hippocampal atrophy with medial temporal lobe atrophy (MTA) of grade 2 (**A**, white arrow), and multiple ischemic white matter lesions with Fazekas scale of grade 1 (**B**, white arrow).

The cranial MRI showed bilateral hippocampal atrophy with medial temporal lobe atrophy (MTA) of grade 2 (Figure 1A), along with multiple ischemic white matter lesions with a Fazekas scale of grade 1 (Figure 1B).

### 2.5 Positron emission tomography/computed tomography (PET/CT)

**Figure 2:**
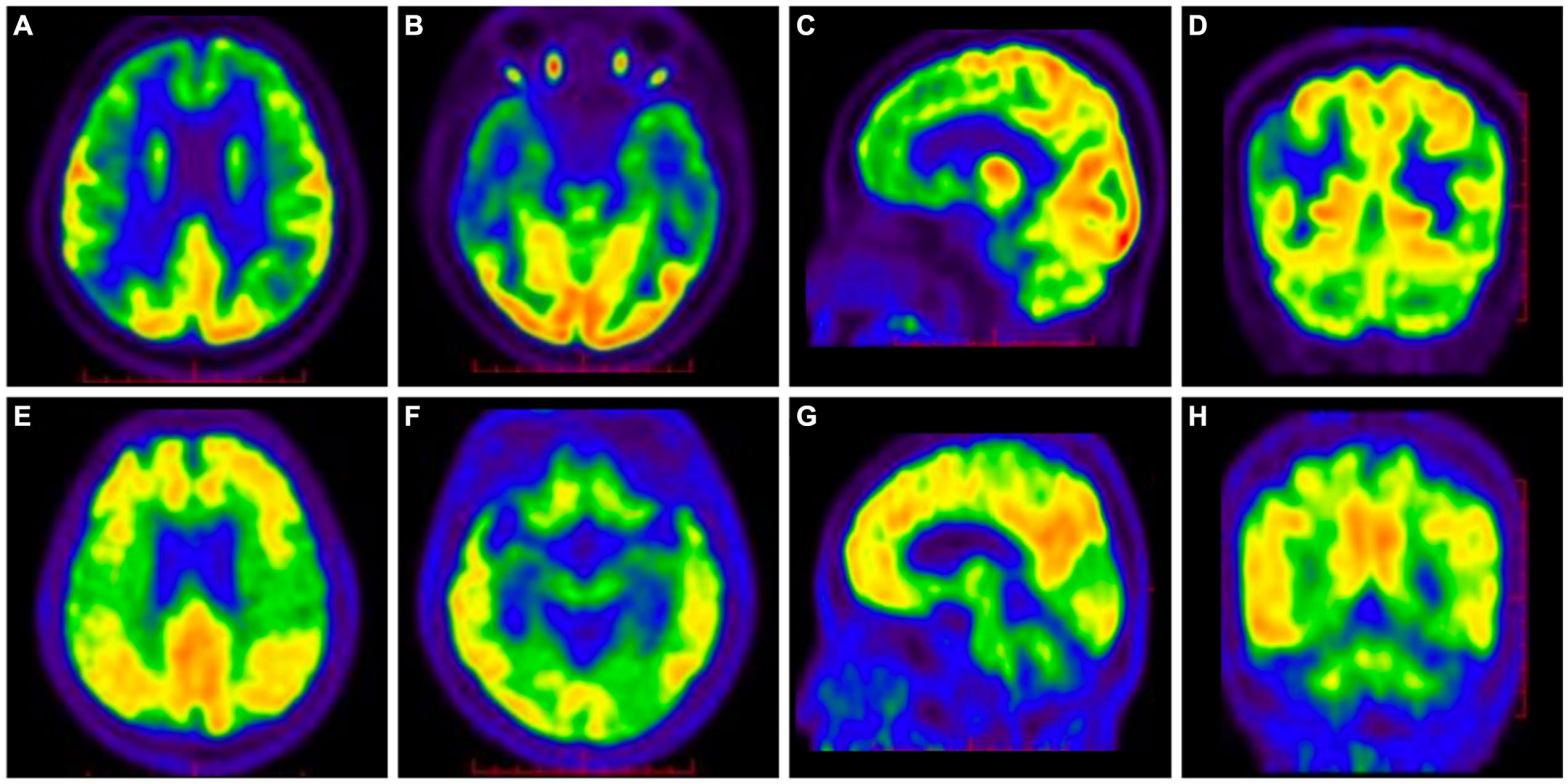
^18^F-FDG-PET/CT showed decreased glucose metabolism and brain atrophy in the bilateral parietal, temporal, and frontal lobes **(A–D)**. ^11^C-PiB-PET/CT showed diffuse deposition of Aβ in the bilateral frontal, parietal, temporal, and occipital lobes, especially in posterior cingulate gyrus and precuneus **(E–H)**.

The ^18^F-fluorodeoxy glucose (^18^F-FDG)-PET/CT scan showed reduced glucose metabolism and brain atrophy in the bilateral temporal, parietal, and frontal lobes (Figures 2A–D), and the ^11^C-Pittsburgh compound B (^11^C-PiB)-PET/CT scan showed diffuse deposition of Aβ in the bilateral frontal, parietal, temporal, and occipital lobes, especially in posterior cingulate gyrus and precuneus (Figures 2E–H).

### 2.6 Genetic testing

**Figure 3:**
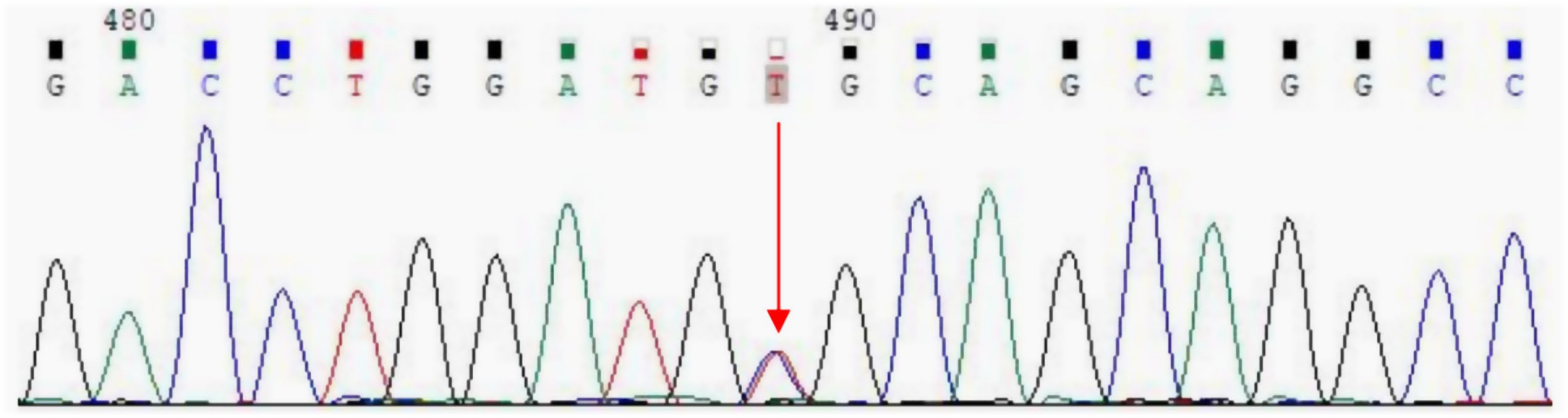
Whole exome sequencing revealed a C-to-T missense mutation of nucleotide 3,755 (c.3755C > T) in exon 25 of the ACE gene, resulting in the change of amino acid 1,252 from alanine to valine (p. Ala1252Val) (red arrow).

Whole exome sequencing was performed to detect exon mutations in all genes, including APP, PSEN1, PSEN2, etc. The result revealed a heterozygous C-to-T missense mutation of nucleotide 3,755 (c.3755C > T) in exon 25 of the ACE gene on chromosome 17q23, resulting in the change of amino acid 1,252 from alanine to valine (p. Ala1252Val) (rs762056936) (Figure 3). This mutation has no reported frequency in the 1,000 Genomes database, with a frequency of 0.00001 in the ExAC database and 0.000004 in the gnom AD database. Since this ACE gene mutation is consistent with the patient’s phenotype, its potentially pathogenic role cannot be absolutely excluded. In addition, her apolipoprotein E (APOE) genotype was ε3/ε3.

## 3 Discussion

We for the first time report the case of a rapidly progressive EOAD patient with a new heterozygous C-to-T missense mutation of nucleotide 3,755 (c.3755C > T) in exon 25 of the ACE gene on chromosome 17q23 (rs762056936). Based on her age, clinical symptoms and the above findings, this patient was consistent with A + T+(N+) (Jack et al., 2018), and not consistent with other causes, including dementia with Lewy bodies, frontotemporal dementia, and Parkinson’s disease, and therefore diagnosed with mild dementia due to EOAD (Rascovsky et al., 2011; Sorbi et al., 2012; McKeith et al., 2017). Subsequent genetic testing identified a novel missense mutation in the ACE gene (rs762056936).

The ACE gene is located on chromosome 17q23, and its primary function is to convert angiotensin (Ang) I to Ang II, a potent vasoconstrictor that narrows blood vessels and increases blood pressure (Nalivaeva and Turner, 2019). Interestingly, ACE has the ability to decrease Aβ level by promoting its degradation and retard its aggregation, deposition and fibril formation, thereby improving cognitive function in AD patients (Hu et al., 2001; Liu et al., 2019).

The most common polymorphism of the ACE gene is the I/D variant of 287-bp in intron 16 (rs1799752) (Yuan et al., 2015), which has been extensively studied in relation to cardiovascular diseases and other health conditions. Studies on different ethnic populations reported that the I/D polymorphism might play a role in the development and progression of AD (Yuan et al., 2015; Xin et al., 2021). However, the results regarding the association between the I/D polymorphism and AD have been inconsistent (Sleegers et al., 2005; Yuan et al., 2015; Achouri-Rassas et al., 2016; Hsieh et al., 2019). Individuals with the I/I genotype showed an increased risk of AD, an enhanced NPI score, and reduced hippocampal and amygdala volumes compared to those with the D/D genotype (Sleegers et al., 2005; Yuan et al., 2015; Hsieh et al., 2019). It was also reported that individuals with the D/D genotype had a significantly increased risk of developing AD and experienced a greater decline in cognitive function over time than those with the I/D or I/I genotype (Sleegers et al., 2005). It is possible that differences in study design, sample size and population characteristics may contribute to these conflicting results from different investigations. Additionally, other single nucleotide polymorphisms of the ACE gene, including rs4343 A/G and rs1800764 T/C, might be linked to AD (Wang et al., 2017). A recent case report from China identified a base change c.A479G in the third exon of the ACE gene, which altered the encoding protein’s 160th codon from aspartic acid to serine, leading to rapidly progressive AD (Ni et al., 2019).

In addition, ACE gene mutations may also be associated with the neuropsychiatric symptoms of AD relating to the frontal lobe (Oliveira et al., 2017; Hsieh et al., 2019). It was shown that carriers of the C allele of rs1800764 had more severe hallucination when their cognition was mildly impaired, and less anxiety when their cognition was moderately impaired. Carriers of the A allele of rs4291 had more severe agitation, disinhibition and irritability, as well as seriously abnormal motor behavior (Oliveira et al., 2017). However, it remains unclear the relationship between the ACE gene (rs762056936) mutation we reported and the neuropsychiatric symptoms of AD at the early stage of the disease, and the underlying mechanism needs further exploration.

More interestingly, when multiple sequence analyses of ACE protein from different species were performed in the Ensembl Database, we found that Ala at the position 1,252 seemed to appear in primate, but is not conserved in other species, whereas, amino acids surrounding this position were mostly conserved. This suggests that this mutation is likely to affect the structure and function of ACE protein, which may only exist in primate. Previous study revealed that lysine to alanine mutation significantly inhibited Aβ oligomerization and aggregation, and reduced its toxicity (Shuaib et al., 2020), which confirmed that the mutations in amino acids affected AD pathology. However, the mutation type of ACE gene we reported here has not been reported in previous studies. The effect of this mutation type on amino acid conservation, the different effects between different species, and its relationship with AD need to be further explored.

This case report has limitations. First, the patient initially presented with delusion and hallucination, however, these symptoms spontaneously alleviated, and the results of scales for neuropsychiatric symptoms were normal. The potential reason needs further investigation. Secondly, her immediate family members refused genetic testing due to geographical restriction, making it difficult to determine whether this gene mutation is familial.

## 4 Conclusion

We report the case of a rapidly progressive EOAD patient with a new missense mutation type in the ACE gene (rs762056936). At present, the relationship between this type of ACE gene mutation and AD is unclear, and the mechanism underlying their relationship remains unknown. We share this interesting case with the main aim of drawing much attention to the potential relationship between this newly discovered ACE gene mutation and EOAD and further focus on exploring the mechanism underlying their relationship.

## Data availability statement

The original data are available from the first author upon reasonable request.

## Ethics statement

Written informed consent was obtained from the patient for the publication of any potentially identifiable images or data included in this article.

## Author contributions

MH: Conceptualization, Data curation, Investigation, Methodology, Writing – original draft, Writing – review & editing. FZ: Conceptualization, Data curation, Investigation, Methodology, Writing – original draft. JQ: Data curation, Writing – original draft. WZ: Funding acquisition, Resources, Supervision, Writing – review & editing.

## References

[ref1] Achouri-Rassas A. Ali N. B. Cherif A. Fray S. Siala H. Zakraoui N. O.. (2016). Association between ACE polymorphism, cognitive phenotype and APOE E4 allele in a Tunisian population with Alzheimer disease. J. Neural Transm. (Vienna) 123, 317–321. doi: 10.1007/s00702-015-1468-3, PMID: 26456241

[ref2] Bondi M. W. Serody A. B. Chan A. S. Eberson-Shumate S. C. Delis D. C. Hansen L. A.. (2002). Cognitive and neuropathologic correlates of Stroop color-word test performance in Alzheimer's disease. Neuropsychology 16, 335–343. doi: 10.1037//0894-4105.16.3.335, PMID: 12146681

[ref3] Buysse D. J. Reynolds C. F. 3rd Monk T. H. Berman S. R. Kupfer D. J. (1989). The Pittsburgh sleep quality Index: a new instrument for psychiatric practice and research. Psychiatry Res. 28, 193–213. doi: 10.1016/0165-1781(89)90047-4 2748771

[ref4] Cummings J. L. Mega M. Gray K. Rosenberg-Thompson S. Carusi D. A. Gornbein J. (1994). The neuropsychiatric inventory: comprehensive assessment of psychopathology in dementia. Neurology 44, 2308–2314. doi: 10.1212/wnl.44.12.2308 7991117

[ref5] Dai M. H. Zheng H. Zeng L. D. Zhang Y. (2018). The genes associated with early-onset Alzheimer's disease. Oncotarget 9, 15132–15143. doi: 10.18632/oncotarget.23738 29599933 PMC5871104

[ref6] Fellows R. P. Schmitter-Edgecombe M. (2019). Symbol digit modalities test: regression-based normative data and clinical utility. Arch. Clin. Neuropsychol. 35, 105–115. doi: 10.1093/arclin/acz020, PMID: 31329813

[ref7] Folstein M. F. Folstein S. E. McHugh P. R. (1975). "Mini-mental state". A practical method for grading the cognitive state of patients for the clinician. J. Psychiatr. Res. 12, 189–198. doi: 10.1016/0022-3956(75)90026-6 1202204

[ref8] Guo Q. Zhao Q. Chen M. Ding D. Hong Z. (2009). A comparison study of mild cognitive impairment with 3 memory tests among Chinese individuals. Alzheimer Dis. Assoc. Disord. 23, 253–259. doi: 10.1097/WAD.0b013e3181999e92, PMID: 19812468

[ref9] Hamilton M. (1959). The assessment of anxiety states by rating. Br. J. Med. Psychol. 32, 50–55. doi: 10.1111/j.2044-8341.1959.tb00467.x 13638508

[ref10] Hamilton M. (1960). A rating scale for depression. J. Neurol. Neurosurg. Psychiatry 23, 56–62. doi: 10.1136/jnnp.23.1.56, PMID: 14399272 PMC495331

[ref11] Hsieh S. W. Liu M. W. Huang L. C. Wu M. N. Yang Y. H. (2019). The impact of angiotensin-converting enzyme gene on behavioral and psychological symptoms of dementia in Alzheimer's disease. Curr. Alzheimer Res. 16, 1269–1275. doi: 10.2174/1567205017666200103114550, PMID: 31902363

[ref12] Hu J. Igarashi A. Kamata M. Nakagawa H. (2001). Angiotensin-converting enzyme degrades Alzheimer amyloid beta-peptide (a beta); retards a beta aggregation, deposition, fibril formation; and inhibits cytotoxicity. J. Biol. Chem. 276, 47863–47868. doi: 10.1074/jbc.M104068200 11604391

[ref13] Hughes C. P. Berg L. Danziger W. L. Coben L. A. Martin R. L. (1982). A new clinical scale for the staging of dementia. Br. J. Psychiatry 140, 566–572. doi: 10.1192/bjp.140.6.566 7104545

[ref14] Jack C. R. Bennett D. A. Blennow K. Carrillo M. C. Dunn B. Haeberlein S. B.. (2018). NIA-AA research framework: toward a biological definition of Alzheimer's disease. Alzheimers Dement. 14, 535–562. doi: 10.1016/j.jalz.2018.02.018 29653606 PMC5958625

[ref15] Johns M. W. (1991). A new method for measuring daytime sleepiness: the Epworth sleepiness scale. Sleep 14, 540–545. doi: 10.1093/sleep/14.6.540 1798888

[ref16] Katsumata Y. Mathews M. Abner E. L. Jicha G. A. Caban-Holt A. Smith C. D.. (2015). Assessing the discriminant ability, reliability, and comparability of multiple short forms of the Boston naming test in an Alzheimer's disease center cohort. Dement. Geriatr. Cogn. Disord. 39, 215–227. doi: 10.1159/000370108, PMID: 25613081 PMC4374652

[ref17] Katz S. Ford A. B. Moskowitz R. W. Jackson B. A. Jaffe M. W. (1963). Studies of illness in the aged. The index of ADL: a standardized measure of biological and psychosocial function. JAMA 185, 914–919. doi: 10.1001/jama.1963.03060120024016 14044222

[ref18] Koss E. Weiner M. Ernesto C. Cohen-Mansfield J. Ferris S. H. Grundman M.. (1997). Assessing patterns of agitation in Alzheimer's disease patients with the Cohen-Mansfield agitation inventory. The Alzheimer's disease cooperative study. Alzheimer Dis. Assoc. Disord. 11, 45–50. doi: 10.1097/00002093-199700112-00007 9236952

[ref19] Liu S. Ando F. Fujita Y. Liu J. Maeda T. Shen X.. (2019). A clinical dose of angiotensin-converting enzyme (ACE) inhibitor and heterozygous ACE deletion exacerbate Alzheimer's disease pathology in mice. J. Biol. Chem. 294, 9760–9770. doi: 10.1074/jbc.RA118.006420, PMID: 31072831 PMC6597817

[ref20] McKeith I. G. Boeve B. F. Dickson D. W. Halliday G. Taylor J. P. Weintraub D.. (2017). Diagnosis and management of dementia with Lewy bodies: fourth consensus report of the DLB consortium. Neurology 89, 88–100. doi: 10.1212/wnl.0000000000004058, PMID: 28592453 PMC5496518

[ref21] Mok E. H. Lam L. C. Chiu H. F. (2004). Category verbal fluency test performance in chinese elderly with Alzheimer's disease. Dement. Geriatr. Cogn. Disord. 18, 120–124. doi: 10.1159/000079190, PMID: 15211065

[ref22] Nalivaeva N. N. Turner A. J. (2019). Targeting amyloid clearance in Alzheimer's disease as a therapeutic strategy. Br. J. Pharmacol. 176, 3447–3463. doi: 10.1111/bph.14593, PMID: 30710367 PMC6715594

[ref23] Nasreddine Z. S. Phillips N. A. Bédirian V. Charbonneau S. Whitehead V. Collin I.. (2005). The Montreal cognitive assessment, MoCA: a brief screening tool for mild cognitive impairment. J. Am. Geriatr. Soc. 53, 695–699. doi: 10.1111/j.1532-5415.2005.53221.x, PMID: 15817019

[ref24] Ni J. Xiao S. Li X. Sun L. (2019). ACE gene missense mutation in a case with early-onset, rapid progressing dementia. Gen. Psychiatr. 32:e100028. doi: 10.1136/gpsych-2018-100028, PMID: 31673674 PMC6802971

[ref25] Oliveira F. F. Chen E. S. Smith M. C. Bertolucci P. H. (2017). Associations of cerebrovascular metabolism genotypes with neuropsychiatric symptoms and age at onset of Alzheimer's disease dementia. Braz. J. Psychiatry 39, 95–103. doi: 10.1590/1516-4446-2016-1991, PMID: 28099631 PMC7111454

[ref26] Rascovsky K. Hodges J. R. Knopman D. Mendez M. F. Kramer J. H. Neuhaus J.. (2011). Sensitivity of revised diagnostic criteria for the behavioural variant of frontotemporal dementia. Brain 134, 2456–2477. doi: 10.1093/brain/awr179, PMID: 21810890 PMC3170532

[ref27] Scheltens P. De Strooper B. Kivipelto M. Holstege H. Chételat G. Teunissen C. E.. (2021). Alzheimer's disease. Lancet 397, 1577–1590. doi: 10.1016/s0140-6736(20)32205-4, PMID: 33667416 PMC8354300

[ref28] Shin M. S. Park S. Y. Park S. R. Seol S. H. Kwon J. S. (2006). Clinical and empirical applications of the Rey-Osterrieth complex Figure test. Nat. Protoc. 1, 892–899. doi: 10.1038/nprot.2006.115 17406322

[ref29] Shuaib S. Saini R. K. Goyal D. Goyal B. (2020). Impact of K16A and K28A mutation on the structure and dynamics of amyloid-β(42) peptide in Alzheimer's disease: key insights from molecular dynamics simulations. J. Biomol. Struct. Dyn. 38, 708–721. doi: 10.1080/07391102.2019.1586587, PMID: 30821624

[ref30] Sleegers K. den Heijer T. van Dijk E. J. Hofman A. Bertoli-Avella A. M. Koudstaal P. J.. (2005). ACE gene is associated with Alzheimer's disease and atrophy of hippocampus and amygdala. Neurobiol. Aging 26, 1153–1159. doi: 10.1016/j.neurobiolaging.2004.09.011, PMID: 15917098

[ref31] Sorbi S. Hort J. Erkinjuntti T. Fladby T. Gainotti G. Gurvit H.. (2012). EFNS-ENS guidelines on the diagnosis and management of disorders associated with dementia. Eur. J. Neurol. 19, 1159–1179. doi: 10.1111/j.1468-1331.2012.03784.x 22891773

[ref32] Starkstein S. E. Mayberg H. S. Preziosi T. J. Andrezejewski P. Leiguarda R. Robinson R. G. (1992). Reliability, validity, and clinical correlates of apathy in Parkinson's disease. J. Neuropsychiatry Clin. Neurosci. 4, 134–139. doi: 10.1176/jnp.4.2.134, PMID: 1627973

[ref33] Wang X. Zhang F. Cui Y. Zheng L. Wei Y. (2017). Association between ACE gene polymorphisms and Alzheimer's disease in Han population in Hebei peninsula. Int. J. Clin. Exp. Pathol. 10, 10134–10139. PMID: 31966905 PMC6965948

[ref34] Wei M. Shi J. Li T. Ni J. Zhang X. Li Y.. (2018). Diagnostic accuracy of the Chinese version of the trail-making test for screening cognitive impairment. J. Am. Geriatr. Soc. 66, 92–99. doi: 10.1111/jgs.15135, PMID: 29135021

[ref35] Xin X. Y. Lai Z. H. Ding K. Q. Zeng L. L. Ma J. F. (2021). Angiotensin-converting enzyme polymorphisms AND Alzheimer's disease susceptibility: an updated meta-analysis. PLoS One 16:e0260498. doi: 10.1371/journal.pone.0260498, PMID: 34818351 PMC8612529

[ref36] Yuan Y. Piao J. H. Ma K. Lu N. (2015). Angiotensin-converting enzyme gene insertion-deletion polymorphism is a risk marker for Alzheimer's disease in a Chinese population: a meta-analysis of case-control studies. J. Neural Transm. (Vienna) 122, 1105–1113. doi: 10.1007/s00702-015-1368-6, PMID: 25596842

[ref37] Zhu X. C. Tan L. Wang H. F. Jiang T. Cao L. Wang C.. (2015). Rate of early onset Alzheimer's disease: a systematic review and meta-analysis. Ann. Transl. Med. 3:38. doi: 10.3978/j.issn.2305-5839.2015.01.19, PMID: 25815299 PMC4356853

